# Surgical management of traumatic C2–C3 subluxation with bilateral carotid artery dissection

**DOI:** 10.1093/jscr/rjaf497

**Published:** 2025-07-14

**Authors:** Vinicius R Ferraz, Marcelo O C S Furlan, Heber M Vieira, Cesar M Yukita

**Affiliations:** Department of Neurosurgery, The Regional Hospital of São José dos Campos - Dr. Rubens Savastano, Rua Goiânia, 345, Parque Industrial, São José dos Campos, São Paulo, 12210-000, Brazil; Department of Health Sciences, Pontifical Catholic University of Paraná, Rua Imaculada Conceição 1155, Prado Velho, Curitiba, Paraná, 80215-901, Brazil; Department of Neurosurgery, The Regional Hospital of São José dos Campos - Dr. Rubens Savastano, Rua Goiânia, 345, Parque Industrial, São José dos Campos, São Paulo, 12210-000, Brazil; Department of Neurosurgery, The Regional Hospital of São José dos Campos - Dr. Rubens Savastano, Rua Goiânia, 345, Parque Industrial, São José dos Campos, São Paulo, 12210-000, Brazil

**Keywords:** C2–C3 subluxation, cervical spine trauma, carotid artery dissection, posterior approach, vascular injury

## Abstract

This case report describes a traumatic C2–C3 subluxation caused by a motorcycle accident following cervical spinal surgery. Traumatic C2–C3 subluxation is an uncommon but serious injury frequently accompanied by considerable neurological deficits. The coexistence of vascular injuries, particularly bilateral carotid artery dissection, complicates the management of such cases. We report a 48-year-old male patient who experienced a C2–C3 subluxation concurrent with bilateral carotid artery dissection and left vertebral artery occlusion as a result of a high-velocity motorcycle accident. The patient underwent surgical stabilization through a posterior cervical approach, followed by endovascular intervention to address the vascular injuries. This case emphasizes the complexities inherent in managing simultaneous spinal and vascular trauma. It underscores the necessity of a customized, multidisciplinary approach to enhance patient outcomes in these challenging scenarios.

## Introduction

Traumatic injuries to the upper cervical spine, particularly at the C2–C3 intervertebral level, are infrequent and are typically the consequence of high-energy mechanisms, such as motor vehicle collisions. These injuries may result in subluxation, vertebral fractures, and potential neurological deficits. The concurrent presence of vascular injuries, such as dissection of the carotid or vertebral arteries, introduces significant complexity to clinical management due to the associated risk of ischemic complications (nonetheless, some cases may present without neurological deficits despite severe vascular injury, as reported by Kiessling *et al*. [1]). This case report delineates the surgical management of a patient presenting with a traumatic C2–C3 subluxation concomitant with bilateral carotid artery dissection and left vertebral artery occlusion, with a focus on the decision-making process and the rationale underlying the selected treatment strategy.

## Case presentation

**Figure 1:**
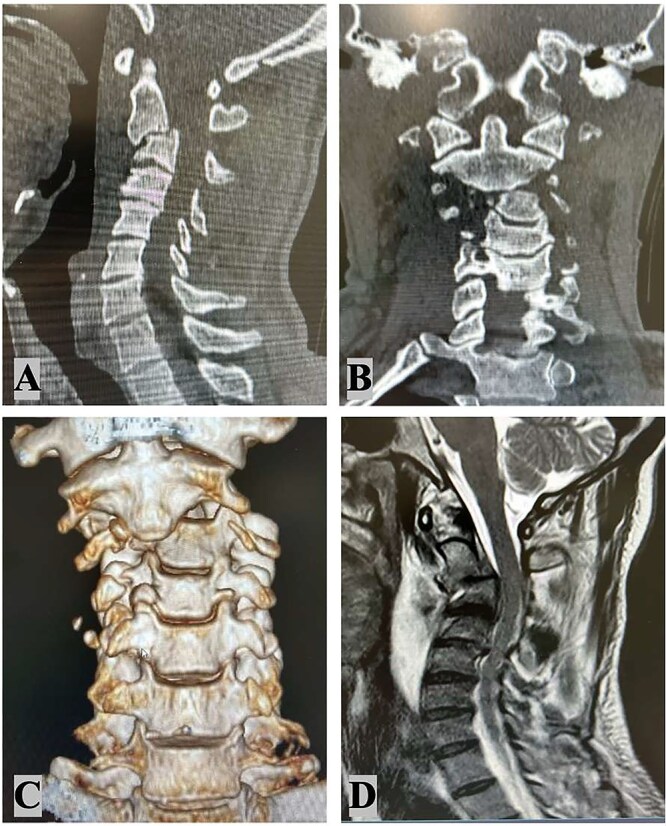
Radiological findings: Cervical spine sagittal computed tomography scan (A); cervical spine coronal CT scan (B) 3D coronal CT scan (C); MRI – C2–C3 subluxation with right lateral listhesis, where the C2 body was positioned on the right lateral aspect of C3 (D).

**Figure 2:**
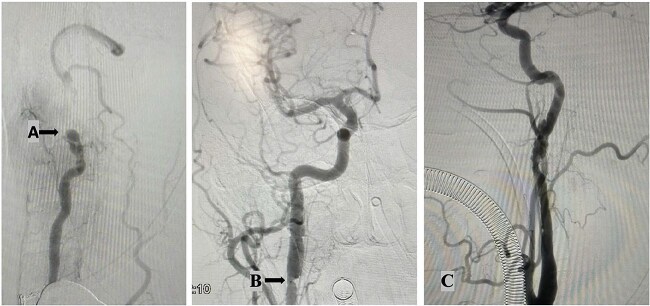
Angiography findings: Total occlusion of left vertebral artery (A); right internal carotid artery dissection shows an intimal flap and thrombi, along with significant hemodynamic changes. These findings are consistent with arterial dissection. (B); left internal carotid artery dissection shows irregularities in the distal cervical internal carotid artery and the left petrous artery, featuring an intimal flap in the lumen and filling defects from thrombi. These changes result from the dissection (C).

A 48-year-old male with no prior comorbidities was involved in a motorcycle accident, colliding with a car at ~50 mph. He was admitted to the emergency department with a Glasgow Coma Scale score of 14, muscle weakness in all four limbs (grade IV), and severe cervical pain [Visual Analog Scale (VAS) 9]. Initial clinical assessment suggested a significant cervical spine injury with neurological involvement. Radiological evaluation included magnetic resonance imaging (MRI), computed tomography (CT) scan (Fig. 1) and angiography (Fig. 2). MRI revealed a C2–C3 subluxation with right lateral listhesis, where the C2 body was positioned on the right lateral aspect of C3, with locked facets on the left. A fracture line was identified through the C3 body at the right pedicle junction, with splayed bone fragments and slight axial rotation. Sagittal imaging showed anterior listhesis of C2 over C3, loss of cervical lordosis, and segmental kyphosis, with an intact ligamentum flavum and no disc herniation at C2–C3. Angiography confirmed total occlusion of the left vertebral artery and dissection of the right internal carotid artery. The injury was classified as AO C-N3-M4 and Levine Edwards Type III, indicating severe angulation and displacement with facet dislocation.

**Figure 3:**
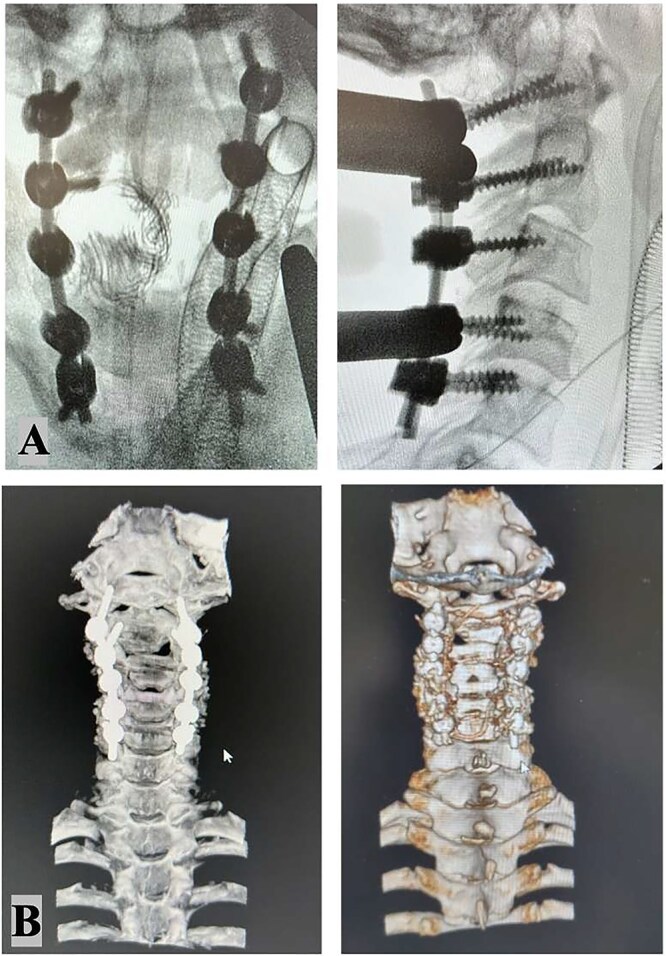
Intraoperative X-ray: Stable fixation and proper alignment of the cervical spine after C1–2–3–4–5 bilateral screw placement (A). Postoperative CT scan imaging confirmed successful realignment and structural integrity (B).

The intricate nature of the injury necessitated thorough evaluation of surgical options, with careful consideration given to systemic, vascular, mechanical, and neurological parameters. The surgical options included external traction, anterior cervical approach, posterior cervical approach, and a combined anterior–posterior approach. Given the presence of bilateral carotid artery dissections, which rendered the vessels exceptionally delicate, an anterior approach was contraindicated due to the heightened risk of iatrogenic vascular injury. Therefore, a posterior-only approach was ultimately selected. The surgical procedure involved unlocking the locked C2–C3 facets on the left side, which required precision drilling and direct manipulation. Realignment was achieved using rod holders as levers, employing counterclockwise rotation of the rods to strategically reposition the lateral masses. To ensure optimal stability with the single posterior approach, fixation was extended to encompass C1, C4, and C5 vertebrae. The patient tolerated the surgical procedure well, and postoperative coronal and 3D CT imaging (Fig. 3) confirmed successful realignment and structural stabilization. Management of the vascular injuries involved starting dual antiplatelet therapy 24 hours after surgery to prevent ischemic or embolic complications, which continued for 6 months. Endovascular treatment of the dissected arteries was performed 15 days following the spinal surgery, addressing the dissection of the right internal carotid artery and the left vertebral artery occlusion.

## Results

Postoperative imaging demonstrated stable fixation and proper alignment of the cervical spine. The patient showed improvement in neurological function at discharge, though specific long-term outcomes were not detailed in the available data. Follow-up care focused on monitoring spinal stability and vascular recovery, with no immediate complications reported.

## Discussion

This case is notable for the rare combination of a traumatic C2–C3 subluxation with bilateral carotid artery dissection and vertebral artery occlusion. The decision to pursue a posterior-only approach was driven by the need to avoid manipulating the dissected carotid arteries, which posed a significant risk of further injury if approached anteriorly. The inclusion of additional levels (C1, C4, and C5) in the fixation construct compensated for the limitations of a single approach, ensuring biomechanical stability stability. The literature on similar cases is limited, but studies such as Amin *et al*. [2], Alexander *et al*. [3] and Mahmoud *et al*. [4] support the use of a posterior approach in complex cervical trauma with vascular involvement. The delayed endovascular treatment of vascular injuries, combined with early antiplatelet therapy, aligns with evidence-based strategies to mitigate ischemic risks, as Merrill *et al.* [5] described. This case underscores the critical need to balance spinal stabilization with vascular management, highlighting the value of a multidisciplinary team encompassing neurosurgery, vascular surgery, and interventional radiology.

## Conclusion

Traumatic C2–C3 subluxation with concurrent vascular injuries represents a challenging clinical scenario. A posterior-only surgical approach can be an effective strategy when anterior access is contraindicated by vascular compromise. Early recognition of both spinal and vascular components, followed by a staged treatment plan, is essential for optimizing outcomes in such complex cases.
